# The training and development process for a world-class female handball player: a longitudinal and retrospective case study

**DOI:** 10.3389/fspor.2025.1655916

**Published:** 2025-10-28

**Authors:** Thomas Haugen, Silvana Bucher Sandbakk, Øyvind Sandbakk, Espen Tønnessen

**Affiliations:** ^1^ School of Health Sciences, Kristiania University of Applied Sciences, Oslo, Norway; ^2^ School of Sport Science, UiT The Artic University of Norway, Tromsø, Norway

**Keywords:** team sport, talent development environment, training recommendations, coaching strategies, longitudinal study

## Abstract

**Introduction:**

Limited information is available regarding the development of young talents who successfully follow the pathway to professional handball. The aim of this study was to retrospectively investigate the longitudinal training and development process of a world-class female handball player.

**Methods:**

An intrinsic case study design was used to capture the quantitative and qualitative aspects related to the training and development process. We used a three-step data collection process and pragmatic analyses of (1) training logs/plans and performance statistics, (2) in-depth semi-structured interviews with three of the player's coaches, and her mother, and (3) systematic quality assurance through interviews to clarify, expand and validate preliminary findings with all informants followed by negotiation among researchers and all key informants, including the player.

**Results:**

The player's exceptional performance level was achieved by a gradual increase in training volume from ∼50 to ∼300 h·y^−^¹ between the ages of 7 and 15, then nearly doubled and plateaued at ∼600 h·y^−^¹ for the remaining career, with handball-specific training and physical conditioning accounting for 300–350 and 250–300 h·y^−1^, respectively. Practically all training was organized, and training in other sports was conducted up to the age of 14. Approximately 80%–90% of the physical conditioning consisted of strength training, while the remaining 10%–20% consisted of endurance training. Key training and development features included early engagement in handball combined with cross-over sampling of various sports during childhood and youth, early introduction and consistent implementation of physical conditioning throughout the career, participation in well-coordinated talent development initiatives, early exposure to adult-level practice and competition, and low injury rate. Key athlete characteristics included a multidimensional sports talent, discipline, dedication, a development-oriented mindset, a strong ability to handle setbacks, and an empathetic nature. Environmental success factors encompassed a sport-enthusiastic family, high-quality training environments, and access to domain-specific expertise.

**Discussion:**

This study provides novel information regarding the training and development process for a female world-class handball player. The findings may provide a framework for how future talents, as well as their coaches, family, training peers, and national sports federations, can effectively coordinate efforts to enhance long-term athletic development and performance outcomes.

## 1 Introduction

Handball is a popular indoor sport played by approximately 30 million people across 130,000 teams worldwide (1). The game is intermittent in nature and involves various motor skills such as throwing, running, jumping, feinting and physical duels. During a 2 × 30 min match, reported mean running distance for female elite players is in the range ∼3,000–5,000 m, and although most of this distance is covered in form of walking and jogging, the match is characterized by frequent rapid accelerations, decelerations, and changes of direction (2–5). Activity profiles vary according to playing position. Backs perform more throws, pivots are more involved in body-contact duels, while wings are involved in more fast breaks compared to the other playing positions (6–8). The ability to produce powerful and accurate shots is crucial to score goals, and mean group values for throwing velocities in the range 72–84 km·h^−1^ have been reported among subsets of female elite players (9–12). To effectively handle the varying demands and become a world-class handball player, technical and tactical skills must be maximized, and a multifaceted profile of position-specific physical/physiological capabilities and anthropometric characteristics is required (5, 8, 13–16). Furthermore, the dueling and explosive nature of handball games and training puts the players at risk of musculoskeletal injuries, predominantly in the knee, shoulder and ankle, requiring systematic injury prevention strategies (17–20). Albeit the popularity of handball, detailed information regarding how high-performing players plan and execute their training to improve performance and reduce injury risk is scarce in scientific literature. Accordingly, there is a need for longitudinal, athlete-centered research in elite handball development.

Limited information is also available regarding the development of youth talents who successfully follow the pathway to professional handball. Several general talent development models have been outlined to assist sporting talents towards a senior career, highlighting the following fundamental features and accompanying sub-factors: (i) athlete characteristics (genetics, birth time of year, anthropometric and physiological predispositions, mental skills, motivational preferences, personality traits, etc.), (ii) environmental factors (training facilities, training peers, family support, coach(es), sponsors, club, federation, sport science, etc.), and (iii) the practice of training (early vs. late specialization, training volume/intensity/frequency, exposure to more high-/adult-level practice and/or competition (i.e., playing “upwards”) during adolescence, etc.) (21–28). However, the general challenge with talent development frameworks is the risk of being overly deterministic, reductionist, and insufficiently flexible to capture the dynamic, multifactorial nature of athlete development. Traditional hierarchical models are based on the notion that early performance holds predictive value (29). In such standardized systems, numerous athletes are de-selected in the progression from one level to the next, leaving very limited opportunities to return. While hierarchical models are based on a strict top-down implementation, the heterarchical talent development model in Norwegian handball is characterized by several developmental initiatives from a range of actors at different levels (clubs, sports schools, regional and/or national sport governing bodies) with mutual constraints and influences. No actor is fully in charge of the holistic talent development process or possess superior instructional authority, leaving considerable responsibility to the players themselves for balancing load and recovery (30).

Norway has been a leading women's handball nation in recent decades (e.g., 25 Olympic, World and European Championship medals since 2000). However, the developmental trajectory from talented child to world-class level has so far not been described in research literature. Overall, more information related to the specific quantitative (i.e., what, and how much) and qualitative (i.e., how and why) aspects of handball training and development is required. The aim of the present study was therefore to retrospectively explore and describe the longitudinal training and development process of a female world-class handball player. Such information is useful for athletes, coaches, sport institutions, sport governing bodies and sport scientists involved in talent development processes, as it supports training optimization, facilitates youth-to-adult transitions, and helps generate hypotheses for future studies.

## 2 Methods

### 2.1 Study design

An intrinsic case study design was applied to capture in-depth information on the development process of a world-class female team handball player. A pragmatic realist approach was adopted, which acknowledges the existence of an objective reality recognizing that our understanding of this reality is shaped by context and the perspectives of those involved. Specifically for this study, we were seeking a comprehensive and balanced exploration of the athlete's developmental path by integrating both objective (e.g., training data, performance statistics) and subjective (interviews) perspectives. Ontologically, we acknowledge the existence of a reality that can be studied; however, we emphasize that its understanding is inherently shaped by the context in which it occurs and by the perspectives of those involved. This means that while phenomena may exist independently, their meaning and interpretation are constructed through social and experiential lenses. Epistemologically, we align with a relational perspective, where knowledge is co-constructed through the interaction between the researcher and participants. Rather than being discovered in isolation, insights emerge through dialogue, reflection, and shared meaning-making within the research process. This permits us to capture both objective historical training data and subjective interpretations to provide a comprehensive understanding of the athlete's development within real-world contexts. The present study design and approach has been applied in several recent studies of best practice training and performance development (31–33).

### 2.2 Case description and informants

The primary case subject is a Norwegian female world-class handball player (age 34 y, height 168 cm, and body mass 62 kg at the time of data collection) whose developmental trajectory from childhood to elite performance provides a unique opportunity to explore the interplay between long-term training, environmental influences, and physiological and psychological factors. She started with organized handball practice at the age of 7 and played for a local and age-specific team for nine seasons, followed by six handball seasons for two Norwegian upper league clubs (16–22 y), four seasons for a French league club (22–26 y) and finally seven seasons for a Hungarian league club (26–33 y). Furthermore, she played for the youth national team (15–17 y), the junior national team (17–19 y) and finally the senior national team (19–33 y), where she was the team captain for the last nine seasons. She also attended a high school with a sport program when she was 16–19 y.

The player's team handball merits include three Olympic medals (one gold, two bronze), five World Championship medals (three gold, two silver), six European Championship medals (five gold, one silver), three Champions League titles, four national upper league championship titles, three domestic (main) cup titles, one gold from Junior World Championship, and one gold from Junior European Championship. Individual awards include International Handball Federation's (IHF) World Player of the Year (2019), All-Star Centre Back of the Summer Olympics (2024), Most Valuable Player (MVP) in World Championship (2017), All-Star Centre Back of the World Championship (2015, 2023), All-Star Centre Back of the European Championship (2018, 2020, 2022), All-Star Centre Back of the Champions League (2019, 2020, 2021, 2022), MVP in Champions League Final4 (2024), MVP in the French Championship (2014), French Championship Best Playmaker (2014, 2016), All-Star Centre Back of the Norwegian Upper League (2011), and All-Star Centre Back of the Junior World Championship (2010).

The data collection involved five key informants:

1. The case subject (from now on referred to as “the player”) was the primary informant, providing training data and personal insights into her experiences, perspectives, and internal processes across age stages/seasons and clubs/teams.
2. The mother of the player (from now on referred to as “the mother”) was the head coach for the local and age-specific teams that the player attended when she was 10–16 y old. The mother was a former handball player at a high national level.
3. The player's head coach (from now on referred to as “upper league coach 1”) during her first two seasons in the Norwegian upper league (i.e., when the player was 16–18 y) for two different teams. At the same time, he was employed as a coach at the high school with a sport program in which the player attended when she was 16–19 y. This coach was a former national upper league player, and at the time of data collection, he was the head coach for the men's national team.
4. The player's head coach (from now on referred to as “upper league coach 2”) during her subsequent four seasons in the Norwegian upper league (i.e., when she was 18–22 y old). He was also employed as a coach for one year (when the player was 16 y) at the same high school as described above. This coach was a former national upper league player.
5. The head coach for the female Norwegian national team (from now on referred to as “national team coach”), in which the case subject played for when she was 19–33 y old. During his 15 y period as a head coach, the Norwegian team won four Olympic (two gold, two bronze), seven World Championship (four gold, two silver, one bronze) and seven European Championship (six gold, one silver) medals.

Altogether, the mother and coaches offered external and professional perspectives on the player's performance development and training dynamics. All informants received written information regarding the benefits and risks of the study prior to signing an institutionally approved informed written consent document to participate in the research project. The Regional Committee for Medical and Health Research Ethics waived the requirement for ethical approval for this study. The ethics of the project was performed according to the institutional requirements. Approval for data security and handling was obtained from the national center for research data (reference number 437719).

### 2.3 Data collection and refinement

The data collection for this study took place between October 2024 and June 2025. A pragmatic three-step procedure was used to collect and refine comprehensive information on the development process of the player:

1. Historical training data, including training records, plans, and developmental logs from the ages of 7–33 were gathered from personal archives and documented by the informants. For the purpose of this study, the training was classified as recorded time in either handball-specific training, physical conditioning or other sport activities. The physical conditioning was in turn categorized as strength/power (in the gym) or endurance training. Reported test results for the case subject were assessed at the Norwegian Olympic Training Centre according to previously described procedures for throwing velocity (34, 35), sprint (36, 37), countermovement jump (38), Keiser leg press (39), bench press (34) and beep test (40). The player's match activities and statistics from the age 16 were retrieved from publicly available websites of the Norwegian, French, Hungarian and European Handball Federation (41–45). To ensure consistent year-by-year comparisons in terms of training and games, season start and end were set to July 1st and June 30th, respectively.
2. A first in-depth interview with each of the key informants was conducted by the first author to document and contextualize the historical data, clarify the remaining details related to the information emerging in step 1, and to provide qualitative insights into training philosophy, considerations, decisions, and environmental factors. Through a structured interview process, training content, planning and execution of specific training at the macro-, meso-, and micro-levels was documented, resulting in a preliminary overview for the ages between 7 and 33. Each interview was performed in-person, lasted approximately 60–75 min (except for the interview with the player, which lasted nearly two hours) and was conducted in Microsoft Teams. The topics and questions differed among the informants according to their specific roles. The player provided personal perceptions, challenges, psychological factors and reflections on the training and developmental process. The mother provided valuable insights regarding the overall training philosophy, specific handball practices, player characteristics and environmental features for the player's childhood and adolescence. Similarly, the coaches offered detailed quantitative and qualitative information from the time in which the player attended their respective teams. Each interview was audio-recorded, transcribed, and approved by the interviewed informant. Formal translation and back-translation from Norwegian to English were performed by the first author and cross checked by the other authors. The interview guides are uploaded as Supplementary Material.
3. Lastly, all informants were actively involved in an extensive review and negotiation process to confirm accuracy and resonance. Triangulation of information from the multiple sources used in this study and follow-up communication *via* videocalls or email were used to refine, expand and validate findings and to ensure that our interpretations reflected their perspectives on the training and development process of the case subject as accurately as possible. Revision of initial reflexive annotations to field notes and reflexive dialogues among researchers and with informants were used to enhance dependability and grounding findings in both researcher reflexivity and participant perspectives.

### 2.4 Analysis

Numerical information (i.e., background information and physical test scores) was systematized in Microsoft Excel (Microsoft Corporation, Redmond, WA, USA). Typical in-season training plans for two consecutive weeks across the player's three upper-league clubs emerged from a combination of typical range in annual training time and number of sessions, time distribution across different training forms and exercise modalities and intensity zones, the amount and type of intensive sessions, as well as typical weekly training volume, training within intensity zones and exercise modalities for typical in-season training weeks across three different time points and contexts (17, 23 and 32 years; Norwegian, French, and Hungarian clubs, respectively). Preliminary summaries of typical training weeks were cross-referenced with historically reported training logs provided by the athlete and further refined through a systematic revision and negotiation process with all informants.

For qualitative information on (a) the practice of training, (b) key athlete characteristics and (c) environmental factors, a systematic six-step procedure inspired by reflexive thematic analysis as proposed by Braun and Clarke (46) was used to analyze the interview transcripts. Step one involved familiarization with the material and initial discussions among all authors. Then, raw themes were identified by level 1 and level 2 coding procedures (step 2) and organized into five, five and three main themes for key training features, athlete characteristics and environmental factors, respectively (step 3). The revision of themes was the result of discussions among authors and a comprehensive structured negotiation process between researchers and participants to assure accuracy and resonance of interpretations (step 4). In the final definition of themes (step 5), contextualization with previous literature on training science was addressed. Ultimately, all authors collectively contributed to writing of the final manuscript, and all presented results were confirmed and approved by all informants (step 6).

## 3 Results

**Figure 1:**
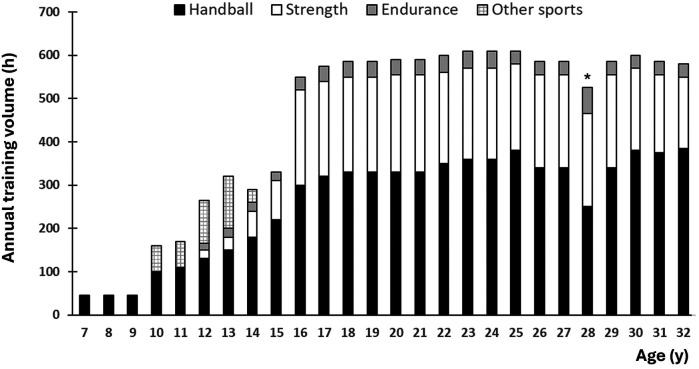
Annual training hours as a function of age. *Reduced handball activity due to COVID-19.

Figure 1 shows annual training hours across age stages. The training volume increased gradually from ∼50 to ∼300 h·y^−1^ at 7–15 y of age. Thereafter, the annual training volume nearly doubled and plateaued at ∼600 h·y^−1^ for the remaining part of the career, with handball-specific training and physical conditioning accounting for 300–350 and 250–300 h·y^−1^, respectively. Approximately 80%–90% of the physical training hours were dedicated to strength training, while the remaining 10%–20% comprised endurance training.

**Figure 2:**
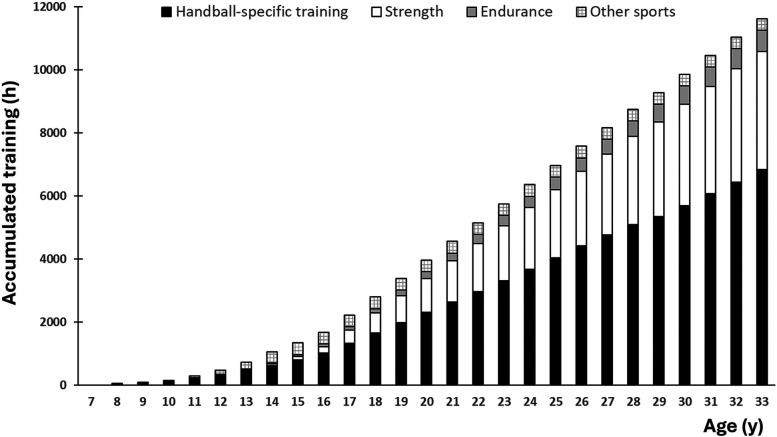
Accumulated training hours as a function of age.

Figure 2 shows accumulated training according to age. The player reached 5,000 and 10,000 h of accumulated training at 20.8 and 29.2 years of age, respectively. Assuming that the player achieved her international breakthrough at the age of 23 (ref. the national team coach's statement in Table 1), she spent approximately 6,000 training hours becoming a world-class player.

**Table 1:** Key athlete characteristics underlying the successful talent development process.

Athlete characteristics	Descriptions and quotes
*Exceptional talent*	The investigated player displayed a multidimensional sports talent from early childhood, and she performed well in both handball, soccer and athletics. She possessed a unique ability to learn new techniques and skills quickly. At the age 10–11, the player's talent for handball started to emerge. Compared to the peers, she exhibited superior technical, tactical and physical skills in form of game understanding, decision making, feints, breakthrough, speed, shooting and change-of-direction abilities. Except for distance shooting, which occurred more frequently during childhood and youth, these qualities remained prominent for the rest of her career.
	The mother: “*X was never among the biggest or strongest players, nor was she the best on the team at the age of ten. When she started playing handball, there were a few others on the team who had already played for some years and had therefore progressed further in their development. However, that gap closed relatively quickly.”*
	The player: “*I was always smaller than my peers, but the difference became even more apparent when I grew older. Because I wanted to be a back player, I had to figure out how to handle the bigger opponents. Based on these constraints, my role as a fast and dynamic playmaker gradually evolved.”*
	Coach 1: “*During my time as head of the handball program for a regional high school, I traveled around to map and identify potential candidates for our program. I remember noticing X at least one year before she was eligible to join us, thinking that we had to make sure she became part of our group the following year. I also recall tipping off the junior national team coach at that time, advising him to keep an eye on her.”*
	National team coach: “*X demonstrated high potential as a playmaker at an early stage. She was a dominant player for a Norwegian upper league club already at the age of 16, something that only a handful of players have accomplished previously. Her key strengths and competencies included technical proficiency, exceptional speed, ability to change direction quickly and vary the tempo. She also possessed strong tactical understanding of the game, particularly in her collaboration with the pivot. These attributes remained hallmarks of her playing style throughout the career.”*
*Disciplined, dedicated and responsible*	All informants in this study highlighted the player's discipline, dedication and responsibility as key features underlying the successful development process.
	The player: “*Although my talent for handball was not particularly evident in the initial years, I found enjoyment in performing well. The competitive element motivated me, and the feeling of mastery was likely present from an early age. It became clear to me that handball was what I enjoyed most, and it was the sport where I wanted to excel. According to my mother, I expressed at the age of nine that I intended to play for the national team.”*
	The mother: “*As X started to cope with the various aspects of handball, she clearly emerged as the most dedicated player on the team. There were several other girls who also were talented, but X was always the one who consistently put in most effort. She demonstrated an exceptional sense of responsibility and was often frustrated with those who did not bother to do the job.”*
	Coach 1: “*She was highly conscientious, both in terms of giving maximal effort and taking responsibility for the team. During her first season in the upper league, she often cried on the bus ride home after defeats as she felt personally responsible. Seemingly, the outcomes of the games were largely attributable to her individual performance.”*
	Coach 2: “*She demonstrated exceptional commitment, never missing a training session or seeking reasons to be absent. Even the few times when injured (e.g., ankle strain), she attended practice and actively contributed to her recovery. Despite a 30–40-min commute each way, she fully engaged in all activities*.”
	The player: “*What I have always found challenging in handball was the constant demand for discipline, responsibility, and the pressure to perform. While these aspects shaped me into the player I became, they also came at an increasing personal cost over time. This was also part of the reason why I finally retired.”*
*Development-oriented mindset and willingness to learn*	According to the interviewed coaches, the player exhibited a strong willingness to learn, seeking advice from trusted people and supporting personnel in her environment. This applied to decisions such as choosing a new club, identifying skills and qualities she needed to improve both on and off the court, training load management, etc. She demonstrated a strong ability to take ownership of these processes herself.
	Coach 2*: “She was highly receptive to learning and feedback and consistently exhibited a high level of awareness regarding which aspects of her game required further development. In these respects, she clearly distinguished herself from the peers. Throughout her adult career, there have been few entirely new elements. Instead, she further developed her key strengths.”*
	National team coach: “*From a technical standpoint, she worked extensively on developing the ability to attack both to the right and to the left, including passing while moving left, as well as various passing techniques to the line player. She also focused on executing effective passes from the central position out to the wing, improving her defensive tasks in terms of positioning and angling, and refining her ability to shoot from the floor. Opponents often gambled on her either attacking predominantly to the right or not shooting at all, so it became crucial for her to develop effective countermeasures.”*
	The player: “*I was fortunate in the sense that most of my coaches over the years shared a common understanding of which aspects of play I needed to improve.”*
*Strong ability to handle setbacks*	Although the player had a successful career, she also had her challenging periods. Her ability to overcome challenges was essential for her development into a world-class player.
	The mother: “*She definitely experienced setbacks in certain periods. In her first upper league season, her team lost all the games and became relegated. In the following season, she became the second-choice player in her position for the new team. Moreover, it took her some years to become an established player on the national team. These situations were somewhat unfamiliar to her. As I had experienced some of the same challenges during my own career, I was able to provide some support, and together we worked through these processes.”*
	National team coach: “*In her first national team championship in 2010, she had limited playing time in the initial matches, and we ultimately decided to send her home and bring in another player for the final games. At the World Championship the following year, her playing time was limited, and in the year that followed, she was not selected for the Olympic Games. During the 2013 World Championship, she played a larger role, but it wasn't until 2014 that she truly made her breakthrough. Elite sport can be unforgiving, and her early years on the national team were challenging. Yet she grew from these experiences—she got her act together, continued working hard, and in the end, her persistence paid off.”*
*Empathetic nature*	The player's empathetic nature was also highlighted by the interviewed coaches. She was always well regarded by her teammates, and this was part of the reason why she was selected as captain of the national team.
	National team coach: “*Her empathetic nature was one of the key reasons why I selected her as team captain, as it is important to understand teammates from their perspectives. However, her empathetic nature was a burden in the beginning of her captain period, as she felt she had to carry the entire team on her shoulders. This was also a process she learnt to work through*.”
	Coach 1: “*She always demonstrated a remarkable degree of warmth and empathy toward her teammates. I cannot think of another example where an athlete who, even at an early stage, combines such a dominant and individualistic presence on the court with a profound concern for others. The best players are often self-centered and egotistical, and there are even persistent myths suggesting that one must be somewhat ruthless to succeed. However, X has clearly demonstrated that it is possible to become the best player in the world while also being a genuinely kind and considerate person.”*

X, the investigated player.

Table 2 shows best physical test scores obtained by the investigated player. Overall, most of the best test results were obtained in her mid-to-late 20s.

**Table 2:** Best physical test scores obtained by the investigated player.

Test	Result	Peak age (years)
Set shot	97 km·h^−1^	24
Jump shot	86 km·h^−1^	26
20 m sprint	2.93 s	28
40 m sprint	5.54 s	26
Countermovement jump	39.1 cm	31
Bench press	71 kg	25
Beep test	13.7 (level)	27
Keiser leg press		
Absolute power	1,408 W	28
Relative power	22.3 W·kg^−1^	28
Absolute force	2,532 N	25
Relative force	38.4 N·kg^−1^	25

Table 3 shows in-season training examples for two consecutive weeks across three of the player's upper-league clubs. During her adult career, typical training weeks in-season consisted of 4–5 handball sessions, 1–2 games and 2–3 physical conditioning sessions.

**Table 3:** Typical in-season training plans for two consecutive weeks across the player's three upper-league clubs.

Day	Norwegian club (17 year)	French club (23 years)	Hungarian club (32 years)
Mon	M: 90 min strength training	M: 90 min strength training	M: 90 min strength training.
	A: 90 min handball	A: 120 min handball	A: 100 min handball
Tue	M:	M:	M:
	A: 90 min handball	A: 120 min handball	A: 90 min handball
Wed	M: 90 min handball	M: Travel	M:
	A: Match (home)	A: Match (away)	A: Match (home)
Thu	M:	M: Travel	M:
	A: 120 min strength training	A: Active restitution	A: 60 min strength + 90 min handball
Fri	M: 90 min handball (easy)	M:	M:
	A: 90 min handball	A: 120 min handball	A: Travel
Sat	M:	M: 120 min strength training	M: 30 min explosive strength
	A:	A:	A: 60 min handball
Sun	M:	M:	M:
	A:	A:	A: Match (away)
Mon	M: 90 min strength training	M:	M: Travel
	A: 90 min handball	A: 120 min handball	A:
Tue	M:	M: 90 min strength training	M: 90 min strength training.
	A: 90 min handball + 30 min endurance	A: 120 min handball	A: 100 min handball
Wed	M: 90 min handball	M:	M:
	A: 90 min handball	A:	A: Match (home)
Thu	M:	M: 90 min strength training	M:
	A: 120 min strength training	A: 120 min handball	A: 100 min handball
Fri	M: 90 min handball	M:	M: 45 min explosive strength
	A: 60 min aerobic endurance	A: 60 min handball	A: 90 min handball
Sat	M:	M:	M: Travel
	A:	A: Match	A: Match (away)
Sun	M:	M:	M: Travel
	A: Match (away) + travel	A:	A:

M, morning session; A, afternoon session.

**Figure 3:**
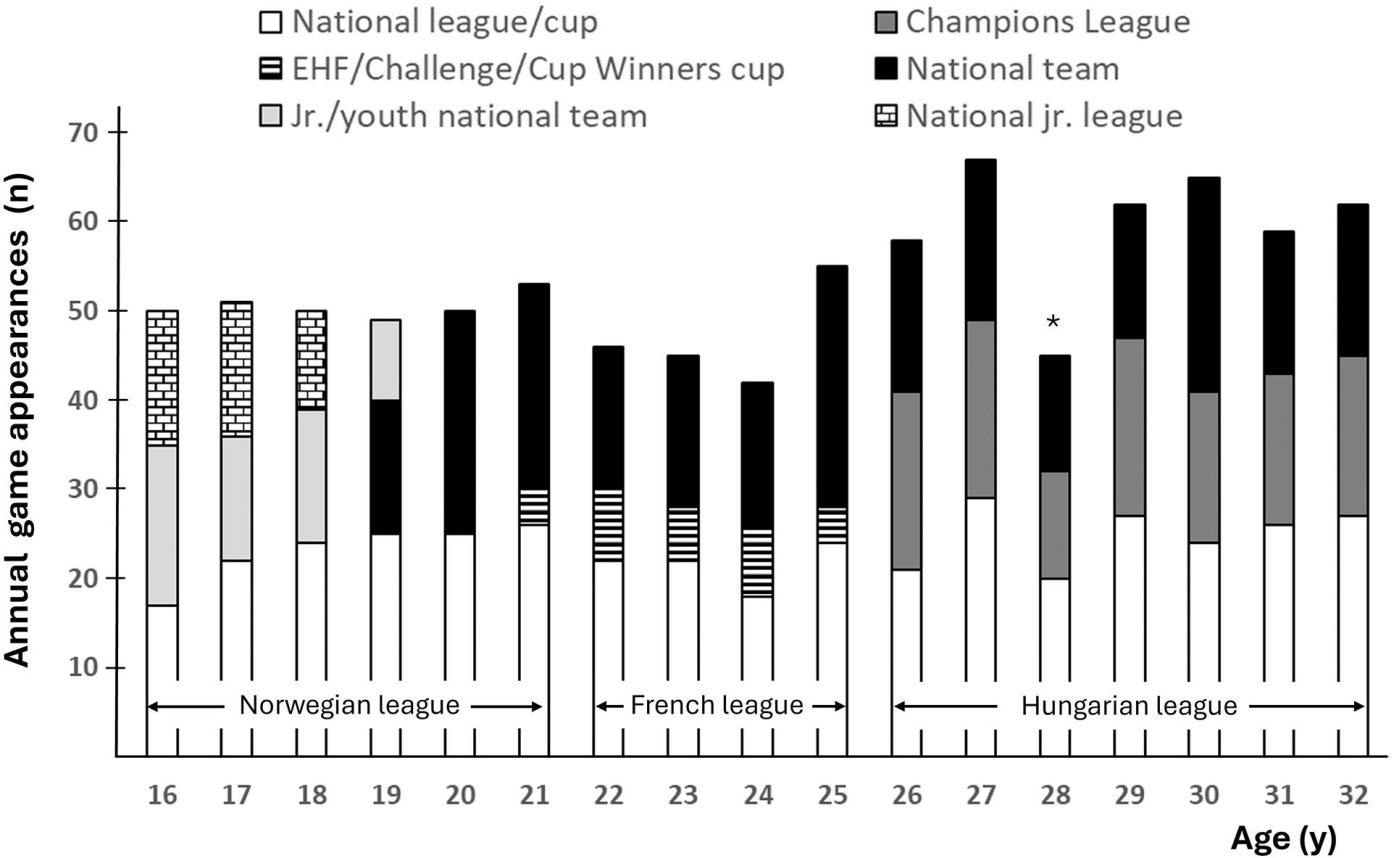
Annual game appearances according to age. *Reduced game activity due to COVID-19.

Figure 3 shows annual game appearances for the investigated player according to age. From the age of 16, she had a total of 822 senior game appearances, in which 143, 112, 298 and 269 games were played in the Norwegian, French, and Hungarian league and for the national team, respectively. National series and cup matches remained stable at approximately 20–25 games per year. Annual game appearances for the national team were mainly in the range 17–23, but slightly higher (25–27) in Olympic seasons. The higher number of annual games at the latter part of her career was mainly due to participation in the Champions League.

The player also attended 30 games for the junior national team (16–18 y), 38 games for the youth national team (15–16 y), and approximately 40 games in the national junior series (14–18 y). Detailed match statistics for childhood and early teens are not recorded due to regulations and restrictions by the Norwegian Handball Federation, but according to both the player and mother, approximately 20–30 games were played in the age-specific and local division system each year in the age 7–15 y.

**Figure 4:**
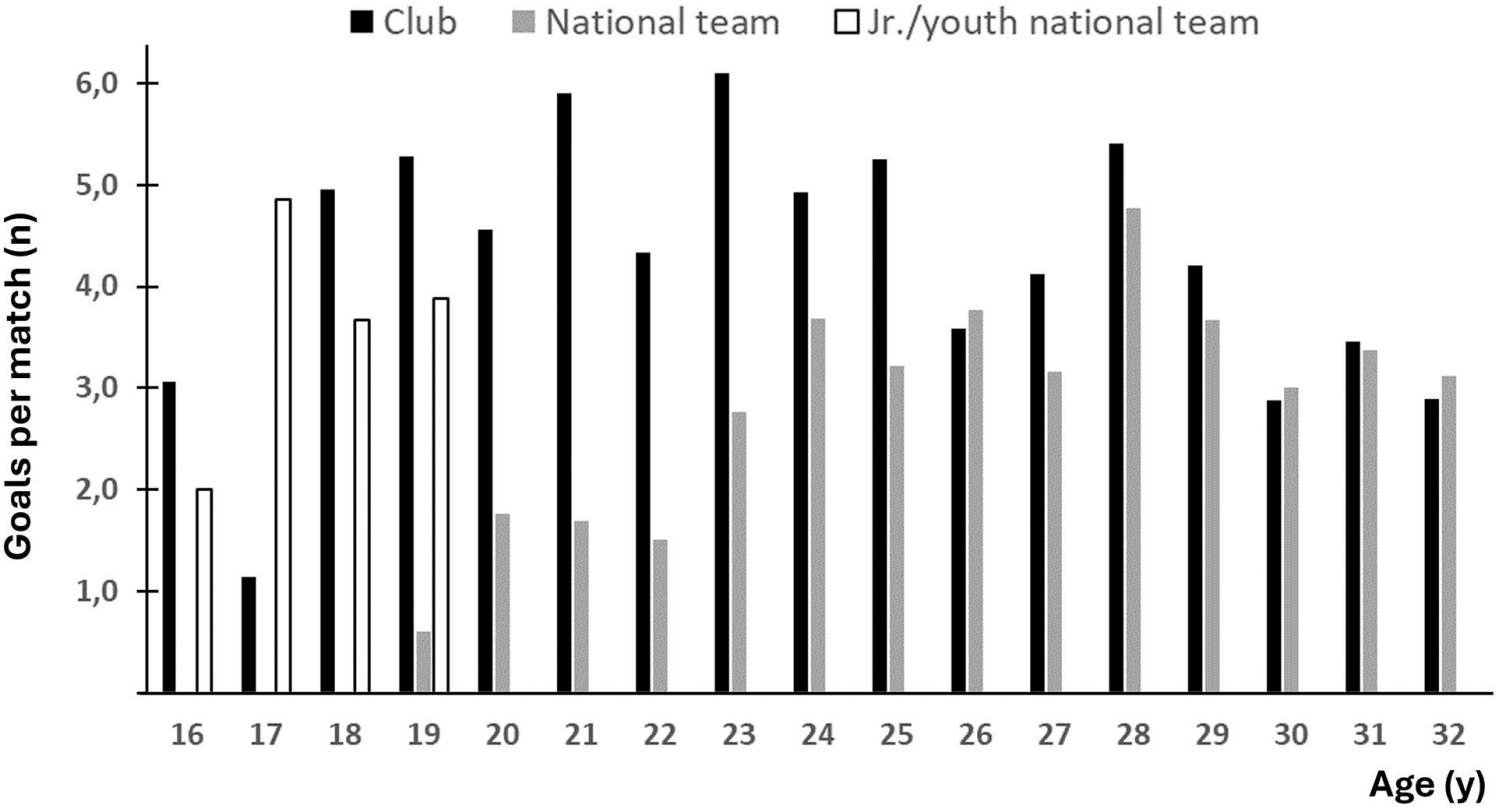
Goals per game according to age.

Figure 4 shows goals per game according to age. From the age of 16, the player scored 3,069 goals in senior games, in which 619, 576, 1,117 and 557 goals were scored in the Norwegian, French, and Hungarian league and for the national team, respectively, meaning an overall average of 3.7 goals per game. She also scored 120 and 103 goals for the junior national team and the youth national team, respectively. According to the player, the slightly lower scoring average during the last three seasons of her career was mainly explained by an even purer and more refined role as a playmaker, both in the club and for the national team, leading to a higher number of assists at the expense of goals scored.

**Figure 5:**
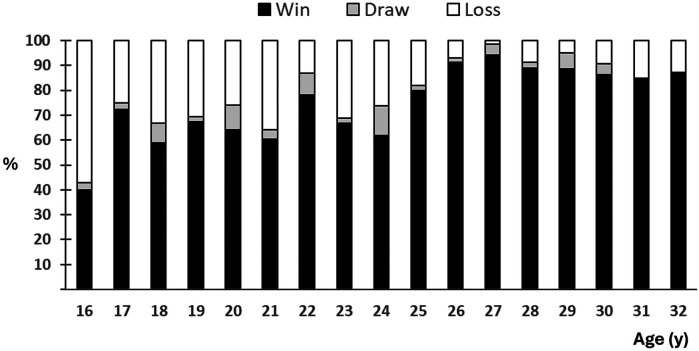
Relative distribution of win-draw-lass across seasons. National junior league matches are not included.

Figure 5 shows the relative distribution of win-draw-loss across seasons. Overall, as the player improved and joined better teams, the winning percent increased gradually up to the age of 26 y for then to stabilize around 85%–90%.

Overall, the player's training and development process was characterized by (1) early engagement in handball, combined with cross-over sampling of various sports during childhood and youth, (2) early introduction and consistent implementation of physical conditioning throughout the career, (3) participation in varying and well-coordinated talent development initiatives during the teens, (4) early exposure to adult-level practice and competition, and (5) low injury rate. For the sake of clarity and readability, the training and development process is presented chronologically in the following sections, allowing the reader to gain a clear impression of the player's progression.

The player's childhood and youth were characterized by versatile activities and cheerful play. According to the mother, the player grew up in a time where outdoor playing in form of cycling, skiing, skating, hockey, rollerblades, etc. were common activities, while digital media and screen time were limited. Practically all training was organized. When the player was 7–9 years old, she performed one weekly handball session (Figure 1) with primary focus on learning basic skills such as passing, receiving, feints and shots on goal. The sessions were characterized by a regular rotation of players across positions and equal playing time during games regardless of ability. When the player was ∼10 years old, her mother became the team's head coach. A considerable part of the mother's handball training sessions consisted of small-sided play such as 3 vs. 2, 4 vs. 3 or 4 vs. 4. This training was designed to ensure frequent ball contact and put the players in situations where they had to make the right choice/decision (e.g., pass the ball to an open co-player). Continuous feedback and corrections were provided throughout these exercises, and there was a significant focus on speed and high tempo during play. They also practiced full-scale (i.e., 6 vs., 6) game scenarios to simulate match conditions.

The player: “*One factor that distinguished us from other teams was our ability to make sound decisions on the court. Our understanding of play was developed at a much earlier stage compared to our opponents”*.

The player also attended soccer and athletics when she was 10–14 and 10–12 years old, respectively (Figure 1), for the following reasons:

The player: “*I started with athletics for the sake of versatility and to become a better handball player. My mother suggested it might be useful for developing my physical abilities such as throwing velocity, speed, power and endurance, and it worked as a form of specialization for handball, just in a different way. Football was more a result of doing well during recess games at school, and since many of my friends played, I started with football mainly for social reasons. Handball was clearly the top priority, while athletics served as a means to improve in handball. Football, therefore, had the lowest priority*”.

During childhood and youth, the player and some of her other teammates occasionally played upwards on sessions and games with the 1-year older team. However, according to the mother, this was not really a bigger challenge as the older team was not on a higher level. Although the player still was the best performer on the court, playing upwards gave her a sense of mastering.

Strength training was introduced for the player's team already when the girls were ∼12 years old. From this period and onwards, strength training constituted an important ingredient of the player's holistic training regime (Figure 1). According to both the player and mother, the early introduction of strength training was ascribed as a potential contributing factor to the low injury rate within the team during puberty.

The mother: “*We started physical training at a much earlier stage compared to most other teams. At training camps, we could meet 15-year-olds who did not even know what a barbell was. We, on the other hand, visited the elite sports center and learned proper lifting techniques, so the girls took physical training very seriously already from the age of 13. The training included both free weights and bodyweight exercises. We had one session per week in the weight room, in addition to shorter bodyweight sessions either before or after handball practice, in order to make the best possible use of the hall time”*.

From age 13–15, the player attended regional talent development initiatives, consisting of monthly training sessions with other selected athletes within the district and one annual tournament in which teams from various regions across Norway came together to compete. These initiatives served as an initial step within the national player development pathway.

The player: “*The regional training camps were arguably a slight step up in terms of level compared to regular club training, but I was among the best players in the group and had no difficulty meeting the performance demands. I regarded the sessions and games as valuable opportunities, as they provided the chance to train and compete with other skilled players. It was also motivating to know that strong performances at the regional level could lead to selection for the national youth or junior teams”*.

When the player was nearly 16 years old and finished secondary school, several crucial decisions were made. Firstly, she had to choose a high school for the three subsequent years. Moreover, because several of her teammates went abroad for student exchange purposes, and the team thereby fell apart, it was time to choose a new club. She therefore switched to a senior upper-league club nearby and at the same time started on a high school in the region which offered a handball-specific program. The underlying considerations were as follows:

The player: “*When I turned 16, I became eligible to play senior-level handball. At that time, the local senior team competed in the second or third division. Therefore, when I was offered a first-team contract from an upper-league club relatively close to where I grew up, I decided to switch. The coaching staff appeared competent, believed in my potential and was willing to invest in my development. Moreover, I felt the environment warm, safe and welcoming. Four other players from my old team switched to the same club, providing me a sense of familiarity and support. The club also offered a strong junior program and a highly competitive junior-league team. I wanted to reach the top level, and joining this new club felt like the right step toward achieving that goal”.*

The mother: “*X was entirely free to choose which high school to attend. However, based on her ambitions, a regular public school was out of the question. We were contacted by the coaches who represented what we considered the best sport-specific high school in the country, and they wanted X to join their program. Attending this high school was prestigious, as nearly all handballers there represented age-specific national teams at that time”*.

Because the high school was not primarily responsible for the talent development process, but rather a supplement to the club activity, the following three focus areas were emphasized: technical training, individual tactical training and physical preparation. During the high-school period (i.e., age 16–19), the player's daily schedule was very hectic, and the training volume was doubled compared to the preceding years (Figure 1). Because her high-school coaches also were responsible for the club training, the load management could be individually adjusted. In this period, her performance level improved even further.

The player: “*Everything intensified during that period. It was important for me to do well, both at school and on the handball court, so there were periods where I was really exhausted. However, being tired was a normal and accepted condition among us students. Overall, I really enjoyed the social life at school, and my body coped well with the large training volume increases”.*

The mother: “*Even today, she does not fully understand how she managed to get through the high-school period, as the days solely consisted of school, training, and homework. In most weekdays, she left 7 AM, attended school, went straight to training afterwards, and was not home until 7 or 8 PM. Still, she considers this period as one of the best times of her life”*.

Coach 1: “*My original plan was to find a mix where she trained and played for both the upper-league team and the junior team. However, because she took the level immediately and became the dominating player on the senior team, I had to change those plans. She proved that she was not too small for senior handball, as she outperformed the opponents with smartness and speed”*.

Coach 2: “*Our coaching team was concerned that the best players should not be overloaded in the pursuit of winning across all levels. Our philosophy was clear: the individual athlete should come first, followed by the elite team, and lastly the junior team. Since X immediately became a central part of the senior team, her involvement in junior team training/games was limited. Handball-specific activity was restricted to a maximum of five sessions per week (e.g., four 90-minute training sessions and one match). She attended three early-morning sessions weekly at school, and we ensured that she had no more than two days per week with double sessions. We also emphasized the importance of maintaining physical conditioning throughout the season, typically including 2–3 weekly strength training sessions. During the summer (off-season), most handball training was replaced with endurance-based conditioning”*.

In her mid-to-late teens, the player had a leading role on the youth and junior national team. These served as important arenas for the player's motivation.

The player: “*The youth and junior national teams were highly valuable and motivating, as it was important for me to have something to strive for. Without these initiatives, I would have felt that the next major milestone as a 14-year-old player was the senior national team, which at that point would feel incredibly far off”.*

At late junior and early senior age, the player was dominant in the Norwegian upper league, and she made her national team debut at the age of 19. The question was where to take the next step, and she undertook careful deliberations throughout this process.

The player: “*We played against a French club in the European Cup. They were not so much better than us, but they played a different style of handball that I found fascinating. So, when I received an offer from them, I felt I couldn't say no. They made it clear that I would get substantial playing time and a central role on the team. There were also a couple of French national team players on the team who encouraged me to join. Altogether, it was a complete package that I found difficult to turn down. Many people questioned my decision to join a second-rated French club. However, I simply found it more interesting to develop my handball skills in France, as opposed to, for instance, going to Denmark, where everything was done in a more traditional fashion, even if the level was slightly higher. The personal development aspect also appealed to me: learning a new language and living in a big city. However, the most important argument was that the national team coach believed it would be a valuable step for my development to join the French club, primarily due to the likelihood of sufficient playing time”.*

According to the player, the transition to French professional handball went very well. Although the overall training load was similar to previous seasons in the Norwegian league (Figure 1), some training-related cultural differences had to be addressed.

The player: “*The training volume was pretty much the same as in Norway. The absence of school/academic responsibilities made the training schedule and routines considerably more comfortable and focused. However, I was not allowed to perform as much physical training as I wanted, as the club typically scheduled only two physical sessions per week. Hence, I discreetly added an extra weekly session at a local gym, following a program provided by a specialist at the Norwegian Olympic Training Center. After a couple of years, I established a good relationship with the club's physical conditioning coach, enabling me to incorporate the training amount I needed. The most notable difference between Norwegian and French players was that Norwegian athletes tended to be far more independent and demonstrated a stronger training culture”.*

The player spent four years in the French club and received several individual awards. At the same time, she established herself as a key player on the national team. However, the desire to win more trophies motivated her to pursue a move to an even more prominent club.

The player: “*I spent four remarkable years at the French club. Each season, I thought we would progress from finishing second or third to finally win the league, but that never happened. Over time, I developed a strong desire to compete in the Champions League and to be part of a team capable of winning major trophies. When the offer came from a world-leading Hungarian club, I felt that this was an opportunity I could not turn down. Both I and several of my national team teammates regarded this club as the ultimate destination—this was the club everyone aspired to play for”.*

Training and playing for the Hungarian club represented yet another step up in terms of quality. The player won many trophies during her seven seasons for the club, including three champions league titles. The total training volume was similar to her previous seasons in the Norwegian and French leagues (Figure 1). However, representing the Hungarian club involved more games (Figure 3) and accompanying travelling.

The player: “*It was highly inspiring for me to join a team that included both prominent athletic performers and strong personalities. The coach told me that most players struggled during their first year at the club, and I was advised to be myself and remain patient. However, due to injuries affecting several other players, I ended up receiving significant playing time during my first season. This early trust gave me a considerable boost in confidence. Having previously played numerous high stakes matches with the national team also contributed to the respect I received from my teammates”.*

As a captain for the Norwegian squad, the player also won many international championship trophies. According to the national team coach, she was a pioneer of a new and modern style of handball.

National team coach: “*Together with two other backcourt players, she revolutionized women's handball. They were shorter players with high tempo, explosiveness, speed, and technical skill—qualities that introduced a new style to women's handball at the time. The traditional model favored taller and heavier players who relied more on long-distance shooting. This trio brought a completely different dimension to the backcourt lineup”.*

In the final seasons of the player's career, the physical conditioning was moderately adjusted to accommodate the physiological demands of an aging body and achieve a better load-recovery balance.

The player: “*The physical coach on the national team made me realize that my legs needed some rest now and then. Since early junior age, I was highly committed to training almost every day, and the strength training typically consisted of two or three weekly 2-hour sessions with heavy weights. During my final 3–4 seasons, I had more days off, shorter strength training sessions and more elements of power and plyometric exercises”.*

The player's career culminated with an Olympic gold medal in her very last match. Her ability to sustain large handball-specific training doses without sustaining severe injuries was highlighted by all informants as a key underlying success factor.

Tables 1, 4 describe key athlete characteristics and environmental success factors highlighted by the informants. Athlete characteristics include a multidimensional sports talent, discipline, dedication, responsibility, a development-oriented mindset and willingness to learn, a strong ability to handle setbacks, and the player's empathetic nature (Table 1). Environmental success factors encompass a sport enthusiastic family, high-quality training environments (i.e., coaches and teammates), and access to domain-specific expertise (Table 4).

**Table 4:** Key environmental success factors.

Environmental factors	Descriptions and quotes
*Sport enthusiastic family*	The investigated player grew up in a family with an affinity for sports. The father was a former soccer player, while the mother was still an active handball player in the player's first years of life. After observing the mother on team practices and games, the player inherited the interested in handball. As the player became older and wanted to go all in for handball, the family schedule was centered around practices, games and tournaments. The importance of strong family support is substantiated by the following statements:
	The mother: “*I believe that growing up in a sports family had a significant impact on her athletic career. To succeed in sports, it is essential to have a family that is both supportive and capable of organizing daily life around athletic commitments. In my view, most individuals who achieve a high level in sports have parents who are deeply involved—sacrificing weekends, attending games and practices, traveling extensively, and providing constant support.”*
	The mother: “*From she was 12–13 y, we reflected on training sessions and matches together. These discussions took place around the dinner table, in the car, at the sports hall—virtually everywhere. Being engaged in sports was an integral part of her upbringing.”*
	National team coach: “*She clearly brought a solid foundation from home with her, characterized by good values, positive attitudes, self-discipline and willingness to do the job.”*
	Coach 1: “*In my opinion, the mother was the single person who contributed most to the player's success.”*
	The player: “*Even though I was her daughter, I never experienced my mother's role as coach as something negative neither for me nor the rest of the team. In fact, my mother was stricter with me, and I had no problem with that. My mother provided me the understanding that achieving success requires hard work, and although you are tired now and then, the job must be done. In many ways, she shaped my mindset.”*
*High-quality training environments*	Throughout the entire career, the player was part of high-quality training environments. In the early teens, the player's age-specific team was among the 2–3 best teams in Norway. This was quite extraordinary, given the low population they represented compared to other teams in the region.
	The mother: “*X was consistently challenged during childhood and youth, as there were quite a few talented girls on her team. Several of them continued playing handball into adulthood at a high national level. Apparently, we did something right. We managed to establish our own playing style and put the players into suitable roles and positions. At the same time, the players felt seen, taken care of and enjoyed themselves. I think it is crucial to find the balance between joy and seriousness for age-specific teams.”*
	In the mid-to-late teens, the player represented an upper league club constituted by the strongest junior group on the women's side in the country. She also represented the youth and junior national team, in addition to attending a prestigious high school with a handball-specific program.
	National team coach: “*In my opinion, she had a nice journey where she had to take her share of responsibility for the teams she played for. During her time as a young handballer, the guidance from her clubs and varying talent development initiatives equipped her with useful tools, preparing her for a professional career at the highest level.”*
	As an adult, the player became part of world-leading club and national team squads. The demanding challenges she faced by teammates and opponents forced her to further develop and refine skills and strategies on the court.
	The player: “*When I joined the French club, I was exposed to a new style of handball. The defensive play in the French league was more unorthodox and focused more on disrupting the opponent—quite different from what I was used to in the Norwegian league. Adapting to this style required me to adjust both tactically and mentally. Joining the Hungarian club represented another step up in terms of handball level, and there were training sessions where I felt that I accomplished nothing. I trained with some of the best players in the world, providing me valuable opportunities to improve and further develop as a player.”*
	Although the player was constantly under the wings of skilled and acknowledged coaches, the national team coach was particularly crucial for her development during adulthood.
	The player: “*I consider myself very fortunate, always being part of high-quality training environments. The varying coaches have contributed to my development in different ways, but the national team coach was particularly important. He selected me for the national team at an early stage and gave me sufficient time to take the next level. He also provided me the opportunity to become a leader (i.e., captain) on the court, a role that many people (including myself) questioned my ability to succeed in.”*
*Access to domain-specific expertise*	As the player progressed and became selected for the youth-, junior- and senior national-team, she gained more access to personnel with domain-specific knowledge within physical conditioning, sports medicine, mental/psychological traits, etc. These experts served as sparring/discussion partners and advisors, offering guidance and support along her developmental path.
	The player: “*The physical coach on the national team was absolutely fantastic. The entire team felt that he possessed all the answers, and he was highly respected among all the girls. He took my physical conditioning to a new and higher level, and I cannot commend him enough. The mental coach also helped me a lot, particularly when I became team captain and had to deal with my conflict-avoidant personality. Overall, access to the Norwegian Olympic Training Center and associated staff was beneficial for my development and made me confident.”*

X, the investigated player.

## 4 Discussion

This is the first study to offer in-depth and longitudinal scientific insight into the training and development process of a female world-class handball player. Her training volume increased gradually from ∼50 to ∼300 h·y^−^^1^ between the ages of 7 and 15, then nearly doubled and plateaued at ∼600 h·y^−^^1^ for the remainder of her career, with handball-specific training and physical conditioning accounting for 300–350 and 250–300 h·y^−1^, respectively. She reached 5,000 and 10,000 h of accumulated training at 20.8 and 29.2 years of age, respectively. Practically all training was organized, and training in other sports was conducted up to the age of 14. During her adult career, a typical in-season training week consisted of 4–5 handball sessions, 1–2 games, and 2–3 physical conditioning sessions. From the mid-teens onward, 80%–90% of the physical conditioning consisted of strength training, while the remaining 10%–20% consisted of endurance training. Key features of the successful training and talent development process included early engagement in handball combined with cross-over sampling of various sports during childhood and youth, early introduction and consistent implementation of physical conditioning throughout the career, participation in well-coordinated talent development initiatives during the teens, early exposure to adult-level practice and competition, and low injury rate. Key athlete characteristics included a multidimensional sports talent, discipline, dedication, a development-oriented mindset, a strong ability to handle setbacks, and an empathetic nature. Environmental success factors encompassed a sport-enthusiastic family, high-quality training environments, and access to domain-specific expertise.

### 4.1 The practice of training

The present study shows that large training volumes were undertaken by the investigated player to reach world-class level in handball. The ∼600 annual training hours from age 16 onwards are comparable to those reported for world-class soccer players and middle-distance runners (47, 48), but considerably lower than previously reported volumes for endurance athletes and a world-leading tennis player (31, 32, 49). However, comparisons across sports have limited relevance, as quantification methods and operationalization of measurable indicators may vary considerably. Moreover, exercise modality significantly affects training load management (50). Handball is characterized by a combination of weight-bearing exercise, physical duels and lower-limb plyometric actions (2–6, 8), which result in high impact forces and, consequently, lower feasible training volumes compared to other, more “gentle” sports. An annual volume of 300–350 h of handball-specific training appeared to represent the player's upper threshold, a plateau reached already in her mid-teens (Figure 1). However, it is important to distinguish training volume from training load in this context, as the performance standard of the teammates and opponents affects the training load considerably. The higher the performance standard, the faster and more intensive play, placing greater demands on the neuromuscular system. Given that the player's volume of specific training remained relatively constant across four different upper-league clubs and three prominent European leagues, it may be speculated if 300–350 h·y^−1^ represents a general upper limit in female top-level handball.

Determining appropriate sport-specific training loads involves careful consideration. On one hand, a substantial volume is necessary to refine technical-tactical skills and develop the requisite physical attributes. On the other hand, high training loads at an early age may increase the risk of overuse injuries, reduce enjoyment, elevate psychological strain, and potentially lead to dropout from sport (21–28). Notably, the absence of unorganized training during childhood and adolescence observed in this study may represent a potential area for further enhancing the overall performance level in handball. While the present player accumulated 5,000 h of total training by 20.8 years of age, previous studies of soccer players have reported reaching similar volumes between ages 14 and 16, with unorganized training comprising the largest portion of total training volume during childhood and youth (51–54). The differences become substantially more pronounced if only specific training is considered (Figure 2). However, the limited availability of handball courts, particularly during the winter months, likely represents a structural constraint. Relevant authorities should therefore consider implementing initiatives to address these infrastructural challenges.

Seemingly, the diversified training undertaken during childhood and youth laid a solid foundation for the player's subsequent career. Although talent development research has revealed considerable variation both within and across sports regarding pathways to elite performance, there is a general and growing trend supporting versatile training and late specialization during childhood. Overall, the present athlete followed a hybrid approach observed in other elite performers, meaning early engagement in the specific sport combined with cross-sport sampling over a relatively extended period (31, 47). However, this does not necessarily imply that participation in other sports at a young age is essential or that it directly enhances the level of expertise ultimately achieved in handball. Regardless of whether an athlete follows a diversified or early specialization pathway, their sport-specific and personal development, as well as their health and well-being, should remain central to any talent development process (55). For optimal developmental outcomes in young athletes, success should be defined in terms of effort, skill improvement, and the cultivation of positive interpersonal relationships, rather than purely in terms of winning (56). Indeed, the present player's handball training with local, age-specific teams during childhood and adolescence was strongly guided by these principles.

Another key success factor in the player's development was the early introduction and consistent implementation of physical conditioning (strength training in particular) throughout the seasons. This training was instrumental in her development into a world-class player, and her distinctive physique became a clear asset on the court. Indeed, the presented test results (Table 2) appear impressive when compared to available data on elite female handball players (10–16, 36–39). Moreover, the player's injury rate during her entire career was very low compared to what previous studies have reported for female elite handball players (17–20). Whether this low injury rate can be attributed to physical conditioning, favorable genetics, or simply luck remains speculative, as many of the player's teammates followed similar conditioning regimes and still experienced severe injuries. Indeed, sports injuries cannot be attributed to a single factor, as their nature is multifactorial (57).

During the teens, the player was selected for several developmental activities initiated by regional and national levels of the Norwegian Handball Federation. Previous studies of other sports have questioned such initiatives, as success at the adolescent or junior level is a poor predictor for long-term success at the senior level (24). However, Bjørndal et al. (58) reported that having youth and junior international experience was strongly associated with the number of matches played at the senior international level in handball. According to several informants in this study, most Norwegian female senior national team players over the past decade have also represented the junior national team, although exceptions do exist. Overall, the above-mentioned initiatives allowed the player to build a personal framework for evaluating and guiding her own athletic development. The comprehensive, regional-level approach to talent cultivation also served as a socializing mechanism, familiarizing her with the expectations and practices associated with elite performance preparation. Consistent with previous findings on talented handball players (59), pushing oneself to the edge of one's capabilities was seen as essential for advancement. Like the present player, these individuals greatly valued the chance to train alongside peers who matched or exceeded their skill level.

The large increase in the player's training volume from age 15–16 (Figure 1) represents a clear violation of one of the most fundamental principles in training science, namely the principle of progression. While she navigated this period without sustaining severe injuries, other handball players typically experience an increase in overuse and injury-related problems at this career stage (59, 60), thereby counteracting long-term development processes. However, the coaches in this case prioritized the player's health over the team's performance needs. It should be noted that the above-mentioned increase in training volume was primarily due to greater doses of physical conditioning, which possibly served as a kind of “vaccine” against potential handball-related injuries. Moreover, although the academic demands were well coordinated with the sporting schedules, the player still experienced the combined pressures as stressful at times, consistent with previous findings on handball players facing similar dual-career challenges (58). Gifted athletes often acknowledge the challenge of balancing a vibrant social life with the demands of a competitive sports career, where education, training, competition, and social life frequently become intertwined (59).

Generally, when several actors are involved simultaneously, the complexity of training activities and competitions increases. According to Bjørndal et al. (58), a talented Norwegian 17-year-old player may regularly relate to more than ten different coaches in various arenas, such as club teams, schools, regional activities or youth/junior national teams. Interacting with multiple coaches across different team environments, each with their own perspectives on athlete development, can lead to conflicting guidance. Many promising handball players have reported experiencing tension due to differing opinions among coaches regarding how to best prioritize and tailor their training routines (58). A high level of communication, cooperation, and coordination among the involved coaches is therefore essential to ensure purposeful and holistic development (61), and this was clearly evident in the case of the present player. The fact that some of her coaches were employed both at the club and the high school was likely beneficial in this regard. While evidence in the literature also suggests that coach turnover or replacement can affect players' injury rate, along with the sheer number of individuals influencing an athlete's training program (61–63), the present player managed to maintain consistency, quality, and strategic alignment of her development process.

Overall, the present player can be considered a successful product of the Norwegian heterarchical talent development model. The starting point was club-based practice and competition, driven by her mother and other parents. As the player advanced through adolescence, she transitioned to higher-level teams and took part in developmental activities initiated by sport schools and the regional and national levels of the Norwegian Handball Federation. Her successful pathway was also marked by exposure to adult-level practice and competition during adolescence. Training alongside and receiving guidance from more experienced athletes appears to provide meaningful developmental advantages for younger athletes. Such interactions can foster the acquisition of technical skills, promote effective training habits, and provide valuable insights into managing adversity and navigating key transition phases (64, 65). Talent development environments that actively promote cross-age training, such as integrating youth athletes into senior-level sessions, can help demystify the demands of higher performance levels and ease the transition toward elite sport (66, 67).

### 4.2 Athlete characteristics

Because no actors in Norwegian handball are fully in charge of the holistic talent development process or possess superior instructional authority (30), each player is left with considerable responsibility for their own development. In the present study, several core personality traits emerged as critical to the player's successful development, and these attributes align closely with existing literature on the psychological characteristics of elite athletes. According to several talent development studies, successful athletes possess high levels of motivation, dedication, confidence, mental toughness, and a strong sense of ownership of the training process (22, 25), traits that align well with those of the present player. Moreover, a development-oriented mindset is a common attribute among elite performers (68). Athletes with this mindset tend to embrace challenges and view effort as essential to mastery. Several times, the present player voluntarily transitioned to higher-level clubs with the intention of enhancing the performance development. Moreover, although the present player had physical limitations in terms of short stature, she compensated by developing exceptional skills in speed, feints, and change-of-direction capabilities to outperform her opponents. Within this setting, talent development in sport is to an increasing extent driven by an asset-based understanding where athlete empowerment represents a key feature (55). Fostering athlete empowerment contributes to an increased sense of autonomy and responsibility in the development process. When individuals are encouraged to set personal goals, evaluate their performance, and actively engage in improvement strategies, it often leads to enhanced self-regulation and a more disciplined approach to training. This sense of ownership, as also observed in the present case, is frequently linked to stronger intrinsic motivation and sustained effort over time (55).

The player's ability to manage the inevitable challenges encountered on the road to elite performance also reflects a psychological characteristic commonly seen in top-level athletes. Rather than being discouraged by adversity, they often use such experiences as opportunities for reflection and growth, enabling them to redefine their goals and support continued development (68, 69). Collins and MacNamara (69) suggest that experiencing a certain level of challenge and adversity is not merely beneficial but fundamental to achieving elite performance. They argue that exposure to such difficulties is an essential part of talent development, highlighting the importance of deliberately incorporating appropriately scaled challenges into the developmental process.

The empathetic nature of the present player is particularly noteworthy, as this trait is less commonly emphasized in traditional performance-focused models but may play a crucial role in team sports such as handball. Empathy contributes to emotional intelligence, which has been shown to enhance communication, cohesion, and leadership within teams (70). It may also support athletes' ability to respond constructively to feedback and foster positive relationships with coaches and teammates. Taken together, the psychological characteristics observed in the present player resonate strongly with prior research identifying key non-cognitive traits that underpin elite sport development. Moreover, the combination of high task orientation (discipline, dedication) with prosocial traits (empathy) and adaptability (resilience, growth mindset) may offer a particularly robust psychological profile conducive to both individual excellence and effective team integration.

### 4.3 Environmental factors

Several environmental factors also contributed to the exceptional performance development, and the mother was the most important single person behind the athlete's success. Parents who consistently offer emotional support through encouragement, praise, and understanding play a pivotal role in nurturing athletes' self-efficacy, intrinsic motivation, and resilience (71). Young talents often view their parents as role models, particularly when parents demonstrate hard work and persistence. In the present case, the player developed an interest in handball after observing her mother during team practices and games. Research emphasizes that a moderate level of positive parental involvement, supportive but not overbearing, is most beneficial (72). Over-instruction or excessive pressure can diminish intrinsic motivation and disrupt the athlete–coach relationship. In contrast, supportive behaviors such as non-directive encouragement strengthen motivation and enjoyment, both of which are essential for long-term engagement and performance (72). Overall, strong family support constitutes a cornerstone in the developmental years of sporting talents (25). Most athletes depend on their parents to help them manage daily responsibilities, including transportation and food preparation, and families are often highly involved in all aspects of their children's handball careers (59). This was also the case for the present player.

The investigated player was consistently under the guidance of skilled and acknowledged coaches. She expressed that all her coaches believed in her potential and were willing to invest in her development, which made her feel safe and welcomed. It remains unknown whether this was also the case for her less-skilled teammates (e.g., substitute players), and it is possible that coaches treat their athletes differently depending on performance level. However, competent coaches are crucial in fostering both athletic progression and personal growth (25, 73). Coaches should not only be technically and tactically proficient but also engage in regular communication with their athletes in an open, clear, and constructive manner. Effectively guiding sports talents also involves preparing them for the rigors and standards of elite senior-level competition by gradually introducing relevant expectations and challenges (73). The success of coaching depends not solely on the coach or the athlete individually, but rather on the dynamic and collaborative relationship they establish. This relationship serves as a crucial foundation for fostering motivation, confidence, satisfaction, and support, ultimately contributing to enhanced performance, a positive sporting experience, and the overall well-being of both parties (73). It has also been argued that the judgements, decisions, and adjustments arising from a well-functioning athlete-coach interplay represent the core of optimizing training quality (74, 75).

The performance level of teammates and opponents represents a critical environmental factor in the talent development process within team sports. In the present study, the player proactively pursued higher-level training environments in line with her advancing performance level. Training alongside or competing against more skilled athletes increases task complexity and fosters accelerated learning and adaptation (22). Through the process of co-adaptation, athletes must acquire new skills or strategies to continuously challenge their opponents with new problems. In essence, they are required to constantly reinvent themselves or demonstrate the capacity to adapt to the evolving strategies employed by their competitors (22). Conversely, training with or competing against less proficient opponents over time can constrain performance development by failing to provide an adequate stimulus for growth. It is therefore crucial to find the optimal challenge level. By deliberately managing the performance standard of teammates and opponents, talent development can be optimized, leading to athletes who are technically proficient, tactically adaptable, mentally resilient, and socially competent. In the present case, the player and her performance team appear to have effectively orchestrated this dimension of the developmental environment.

Besides coaches and teammates, the support staff forms a highly important resource within a talent development environment (76, 77). Having access to educated helpers who can provide an excellent standard of support in everyday life and especially in challenging times, promotes athletes' chances of successful development (78–80). The player specifically emphasized the support she received from a specialist at the Olympic training center in modifying her physical training, as well as from a coaching expert who helped her clarify her thoughts and define her role as captain of the national team. The findings of this study further underscore the critical role of effective collaboration and communication among all stakeholders, including the athlete, coaches, medical staff, club, educational institutions, and national federation, in facilitating long-term athlete development. In general, the training and development process observed in the present study align with key principles presented in existing talent development frameworks (21–28, 30, 52, 59).

### 4.4 Methodological considerations

Some study limitations need to be addressed. Whereas previous research on talent development typically relies on either quantitative or qualitative methods (22), this study sought to integrate both approaches. For the qualitative component, we evaluated the study using Tracy's eight key markers of quality (81): (a) worthy topic, (b) rich rigor, (c) sincerity, (d) credibility, (e) resonance, (f) significant contribution, (g) ethics, and (h) meaningful coherence. As outlined in the introduction, the topic is considered worthy as it addresses important questions concerning the training and development process of a world-class handball player. We sought to ensure rigor, sincerity, and credibility by combining established methods of data collection that generated rich qualitative and quantitative material from multiple perspectives; conducting thorough review and negotiation processes to confirm accuracy and resonance; and maintaining transparency regarding methodological procedures and limitations. We regard the study as making a valuable contribution to both scientific knowledge and applied practice. Ethical considerations included obtaining formal approval for the research and adopting respectful practices to safeguard a collaborative relationship between researchers and participants. Although all participants were anonymized, they were aware that they could potentially be identified based on the information presented in the study, and they consented to participate under these conditions. Meaningful coherence was obtained by adhering to the intended project design, with alignment of study aims, epistemology, conceptual frameworks, methodology, and presentation of findings.

A common challenge in case studies focusing on training content is ensuring the accuracy and validity of the data. Nonetheless, elite performers and teams generally maintain systematic and precise records of training activities, supporting the use of training logs as reliable primary or secondary sources in scientific investigations of training characteristics (48, 49, 82, 83).

To mitigate bias and misinterpretation, we incorporated triangulation across methods, data sources, and investigators. We observed a high degree of consistency between the player and the respective coaches in terms of retrospective data related to the practice of training, athlete characteristics and environmental factors. However, it remains possible that parts of this story may have been nuanced if other informants (e.g., teammates or other coaches) were included. Moreover, accurately assessing training load remains challenging due to the intermittent nature of handball, and recorded time is not necessarily the most appropriate approach because of recoveries and fluctuations in intensity. Indeed, more detailed information on the training load from handball-specific sessions could potentially contribute to a more comprehensive understanding of an athlete's career trajectory. Another caution is related to the large variation in pathways to success (21–28), as several roads may lead to Rome. Many handball players have engaged in similar training regimens during childhood and adolescence without reaching the same level of success. Therefore, the findings should be interpreted carefully, as no causal relationships can be established.

The authors' professional background, including former and/or present leading positions within the Norwegian Olympic Federation and acknowledged high schools with a sports program, is both a strength and a potential source of bias. Collectively, the research team has over three decades of experience working with elite athletes and talents, including world-class handball players. This expertise provided unique access and contextual understanding, enhancing our capacity to collect and interpret the data. However, we also acknowledge that this familiarity may have influenced our interpretations.

## 5 Conclusions

This case study provides novel information regarding the training and development process for a world-class female handball player. Here, we have presented quantitative training data from early childhood to end of career, as well as qualitative data where key success factors were identified. Between the ages of 7 and 15, the annual training volume progressively increased from approximately 50–300 h. In the subsequent season, this volume almost doubled, leveling off at ∼600 annual training hours throughout the rest of her athletic career, with handball-specific training and physical conditioning accounting for 300–350 and 250–300 h·y^−1^, respectively. Most of the training was organized, and she participated in multiple sports until the early teens. Throughout her adult career, typical in-season weekly schedules included 4–5 handball sessions, 1–2 matches, and 2–3 physical conditioning sessions. From her mid-teens, 80%–90% of the physical conditioning consisted of strength and power training, while the remaining 10%–20% was dedicated to endurance training. Key features that contributed to a successful training and talent development process included early engagement in handball combined with cross-sport sampling during childhood and youth, the early introduction and consistent implementation of physical conditioning throughout the athlete's career, participation in well-coordinated talent development initiatives during the teenage years, early exposure to adult-level practice and competition, and a notably low injury rate. The athlete's personal characteristics also played a crucial role, including a multidimensional sports talent, strong discipline and dedication, a development-oriented mindset, the ability to handle setbacks effectively, and an empathetic nature. Finally, environmental success factors such as a sport-enthusiastic family, access to high-quality training environments, and domain-specific expertise were essential in supporting long-term development and performance at the world-class level. Overall, the player's development reflects the success of the Norwegian heterarchical talent system. The training implemented, along with the strategic decisions, priorities, and reflections made by the player and her performance team may serve as a potential model for how upcoming talents, as well as their coaches, families, training partners, and national sport organizations, can operate and collaborate to optimize long-term performance development. The presented findings have also generated novel hypotheses for future studies dealing with talent development processes.

## Data Availability

The datasets presented in this article are not readily available because, in order to protect the anonymity of the informants, the transcribed interviews cannot be made publicly accessible. Further enquiries should be directed to Thomas Haugen, thomas.haugen@kristiania.no.
