# Supplementary material for: The training and development process for a world-class female handball player: a longitudinal and retrospective case study
Source: Front Sports Act Living. 2025 Oct 28;7:1655916. doi: 10.3389/fspor.2025.1655916 (PMC12602410; doi:10.3389/fspor.2025.1655916)
Supplement: Supplementary file 1 [file Datasheet1.docx]

**Interview guides**

**The player**

Could you give a general description of your childhood and upbringing in terms of physical activity? Did you grow up in a sports-interested family? Were you physically active with your family (hiking, outings, playing with siblings)? Were your parents former athletes?

Can you describe what your training sessions were like in the early years at your local club? What characterized the environment? What was emphasized — performance, a positive atmosphere, results, other things? Were your teammates mostly classmates? Did you spend time together socially outside of handball?

What made you continue with handball? (Social reasons? The competitive/performance aspect? Other reasons?). Looking back, how would you describe yourself as a player compared to others on the team at that time? Did you already have some of the same qualities you had as an adult Were you the talented star of the team also as a youngster, or did it take some years before your talent envisioned? How was your motivation and training/performance attitude compared to your peers at that time? Were there other personal characteristics in which you differed from the other peers? Did you train or play matches with older players, or always with peers at the same age?

Did you train or play handball outside of team practices? Did you engage in a lot of unorganized training at home or school? If so, can you describe how this individual training was carried out?

Did you have good access to training facilities (hall time), or was this a limiting factor?

Your mother was mostly your coach while you played for your local club (except at the very beginning). How was it to have your mother as a coach? Did you ever find it problematic? What role has your mother played in your career?

Eventually, you also started with football and athletics. What led you to take up these sports? Could you describe that training? Did you perform well in these sports? How did it rank in priority compared to handball?

As players grow older, the number of matches increases, and performance and results become more emphasized. How did you and your team perform as you aged? Did you perform well compared to other teams in the region? Did you experience any setbacks or stagnation?

When transitioning from elementary to secondary school, many athletes drop out. How did you experience this transition? Did many on your team quit? Did this make it difficult to keep the team?

Did you experience puberty and physical changes as challenging in relation to handball, whether in terms of performance or injuries?

What made you eventually decide to leave your local club?

What made you decide to join a national upper-league club?

At the same time, you started at a high school with a sports program. Why did you choose this school? How did you experience the transition — new club, new school? Were the changes overwhelming, or did it go smoothly?

How did you experience the changes from a sporting perspective — the increased training load and practicing with more skilled players? Did you feel you could keep up with the level and improve by switching clubs? How did you manage the logistics — did you go straight from school to practice, or go home first to eat and rest?

How was the communication between the school and club in terms of managing your training load? How was training divided between school and club?

During this period, your training volume increased significantly. How did your body handle that in terms of illness and injury?

How was it dealing with multiple coaches (school, club, national youth teams)? Did conflicting advice ever become frustrating, or were the coaches aligned in their communication?

Were there aspects of your game that you focused on to develop as a player? Did you get to work on your individual needs, or did that often conflict with team needs?

Some coaches said you had challenges with pacing and tempo control at the time (too much speed). What did you do specifically to improve in that area?

How did you prioritize school in relation to handball?

At sports high schools, some athletes continue and succeed while others drop out. Why do you think you were among those who succeeded, while some talented peers quit?

You only stayed one year at this upper-league club. Why did you choose to leave?

What role has Coach 1 played in your development — both as your club coach and at school?

What factors led you to choose another upper league club?

How did you experience that transition? Was the training of even higher quality? Were there major changes in training volume or intensity?

You played in the national upper league already at age 16 — something very few have done. Have you reflected on why you became so good so early? Do you think you would perform just as well today in the same league at 16, or has the league become tougher for younger players?

Did you live at home, or move out while attending high school and playing in the elite league?

What impact has Coach 2 had on your career?

What made you decide to leave the Norwegian elite league?

Coach 2 mentioned that many were surprised when you moved to a French club. What led to that decision?

How did you experience that club change? How was it to move and live on your own? Were there major changes in training and coaching compared to the Norwegian league? Were there specific things you focused on to improve as a player?

You were awarded Player of the Year in France for three seasons, so it appears you thrived and developed well there. Why do you think you succeeded?

Why did you leave the French club after a few years?

Why did you choose to join a Hungarian club?

How did you experience that move? Were there major changes in training (volume, quality) and coaching compared to France? Were there new challenges in terms of culture, language, living alone in a new country, new coach, teammates, etc.? What were your main areas of focus during this period?

Training data shows your training volume declined slightly toward the end of your career. Why?

You won many titles with the Hungarian club. Why do you think you succeeded there?

How has your role on the court evolved over time, both in defense and offense?

You retired last year. What made you decide to stop? Was it physical limitations, motivation, mental factors, other things?

Let’s talk about your time with the national team. You played for both the junior and senior teams. How did you experience these arenas, both athletically and socially? Did they help you develop as a player?

In your first major tournament for the senior national team, you were sent home and replaced after the initial round. It took several years before you established yourself on the senior national team. How did you experience these troubling years? What helped you get through that period and eventually succeed?

Coach 2 mentioned he was surprised you eventually became the national team captain, as he had experienced you as conflict-averse earlier in your career. What personal development led to you taking on that leadership role?

How did you experience the support system around the national team? Was it a great help? Not just the head coach and assistants, but also the physical trainer, mental coach, medical staff, etc. What role did this team play in your development?

If you were to summarize your career — what were the most important reasons you became a world-class player?

If you were to highlight a few people who had a major impact on your career — who were they, and why?

You participated in both regional and national talent development initiatives and projects during your teens. How did you experience those initiatives? Do you feel they contributed to your development as a player?

Would you characterize yourself as a successful product of the Norwegian talent development model, or did you succeed because you went your own way and took personal responsibility for your development?

That’s all the questions I have. Is there anything you would like to add—something I haven’t asked about, but you feel is important to mention?

**The mother**

Can you give a general description of X’s childhood and upbringing in terms of physical activity? Did she grow up in a sports-interested family? Was she physically active with the family (hikes, outings, playing with siblings)? Were you (the parents) former athletes?

What led X to eventually start playing handball?

Given that X grew up in a sports- and handball-oriented family, what role do you think this played in her handball career? What do you think was most important in that regard—your knowledge as a former handball player, or the practical support like driving, cooking, doing laundry, volunteering, etc.?

Was the family’s schedule at home largely centered around sports?

Was it mostly the team’s organized practices, or did the girls also engage in unorganized training on their own?

How was X as a handball player in her childhood compared to the others? Was she better, more eager, more motivated, or was it a homogeneous group?

Can you describe what training was like in the early years at the local club? What characterized the training environment? What was the focus—performance, a good social atmosphere, results?

What motivated X to continue with handball? Was X dependent on her friends to go to practice? Did she play upwards (i.e., training with older peers)?

Did she already have many of the same attributes on the court as a child that she later had as an adult (e.g., small in stature, strong feints, high tempo)?

According to X, the team became better than others at making good decisions and choosing the right options on the court—in other words, they developed better game intelligence. Do you agree with this? Could you elaborate?

What kind of training exercises did you use to help the girls become better at making smart decisions?

Did you have sufficient access to training facilities (hall time), or was that a limiting factor?

Did you ever find it problematic to be both X’s mother and her coach?

At some point, she also started football and athletics. What made her start those sports?

How did X and the team perform on the handball court as they got older? Did they experience setbacks or stagnation, or was it mostly smooth progress?

You lived in a small place, so it seems quite remarkable that a club from such a small community could have an age-group team among the best in Norway. Has this happened before in this club? The fact that you coached a team from a small town to be among the best nationally indicates you were a good coach, doesn’t it?

Was your training philosophy something you developed over time, or did it come from your own time as a handball player?

As children grow older and start secondary school, the dropout rate in sports increases. How did you experience the transition from primary to secondary school in the team you coached in terms of dropout? Did many of the players quit? Did this eventually make it difficult to keep the team?

What made X eventually leave the local club and join a senior upper-league club? How involved were you in that decision? Was it entirely her choice, or did she discuss it a lot with you?

Why did she choose to attend the high school with a sports program? To what extent were you involved in that process and decision?

Did you find that puberty and physical changes were challenging for X in terms of handball, whether in terms of performance or injuries?

If you were to summarize—what do you think made X become a world-class player?

That’s all the questions I have. Is there anything you would like to add—something I haven’t asked about, but you feel is important to mention?

**Coach 1**

Can you briefly describe your career as a player and coach?

Can you elaborate on your general philosophy regarding talent development? How should one approach the process of guiding a young talent to become an international-level player as a senior?

X started training with you—both in an upper-league club and at a high school with a sports program—when she was around 16 years old. Did you think at that point that she had the potential to become a world-class player?

How would you characterize X as a handball player when you first started coaching her (compared to her peers)?

What were her technical and tactical abilities? Did she already possess many of the same qualities she later became known for as a senior player (being small in stature, strong in one-on-one situations, fast-paced)? What were her physical attributes like (strength, explosiveness, speed, endurance)? What about her mental traits and personality? Motivation, ambition, competitive instinct, independence, eagerness to learn and improve? How did she function within the group?

X came from a local club where she played at the youth level. Did you have a specific strategy for how to integrate her into your upper-league team, both in training and in matches? Was she “thrown to the wolves” right away, or did she play/train with equally aged peers? Did you notice whether X found it intimidating to train or play matches with much older and physically stronger players, or did she seem unfazed by it?

From what I understand, X earned a regular spot on your upper-league team at the age of 16. How common is it for players that young to compete at the highest senior level in Norway?

What were the main areas of focus in your work with X to help her develop as a handball player? Did you emphasize technical/tactical, physical, mental, or relational aspects? Could you explain how you distributed X’s training load between school and club? How was the coordination between these two arenas?

In the year when X started at the high school with a sports program and also joined an upper-league club, her training volume increased significantly. Do you recall whether she experienced any challenges related to this increased training and match load, such as illness or injury?

X’s mother eventually became part of your coaching staff. What role do you believe she played in X’s development?

What impact has the high school had on X’s development?

Would you say that X became successful because of, or in spite of, the Norwegian model for talent development? Has she followed the traditional path, or created her own?

What are the most important reasons why X developed into a world-class player?

That’s all the questions I have. Is there anything you would like to add—something I haven’t asked about, but you feel is important to mention?

**Coach 2**

Can you tell us a bit about your own career as a player and coach?

Can you give a general description of your training philosophy, specifically how to develop a young talent to succeed at senior level?

X trained and played under your leadership for four seasons. What were your initial thoughts when you observed her at those first training sessions? Did you think right away that she might become a future world-class player?

How would you characterize X as a handball player at that time in terms of:

a) Technical and tactical abilities

b) Physical attributes (strength, speed, endurance, etc.)

c) Mental aspects (motivation/dedication, ambition, competitive instinct, focus, independence, etc.)

d) How she functioned socially within the group

In what areas did she stand out compared to the other players?

Did you have a specific plan for how to train and develop X to optimize her progress? Were there particular aspects you focused on?

Can you describe how X developed over the years while you coached her? Was her progress consistent, or were there periods of setbacks and challenges?

You also coached X during her first year at the high school. How was the training coordinated and distributed between school and club? During that year, X’s training volume increased significantly. Do you remember whether she experienced any challenges related to this increase in training and match load—such as illness or injury?

How did X handle working with multiple coaches at the same time (school, club, national team)? Were there any conflicts between individual development needs and the team’s needs? These are factors that can often cause stress. How did she manage these situations?

X started playing for the national team while you were still coaching her at club level. However, it took her a few years to fully establish herself at that team. How would you describe that period for her? How did she deal with these challenges? What was the dialogue like between you, X, and the national team staff in terms of supporting her development?

In your opinion, what are the most important reasons why X became a world-leading handball player?

Eventually, X chose to move to a French club. What was the reasoning behind this decision? What were your thoughts on that choice?

That’s all the questions I have. Is there anything you would like to add—something I haven’t asked about, but you feel is important to mention?

**The national team coach**

For the past 15 years, your job has been to identify top-level talents, develop them, and build them into a well-functioning team. What is your philosophy for developing young and promising talents into national team players?

Based on your answer to the previous question, one could summarize your role as follows: In addition to planning, organizing, and running training sessions, camps, and matches, you’re also responsible for managing the resources around you—assistant coaches, strength and conditioning staff, mental specialists, medical personnel, etc.—so that the players can benefit as much as possible on their journey. Would you agree with that summary?

You have a large support team with many experts involved. Can it sometimes become a case of “too many cooks in the kitchen,” with conflicting messages being communicated to the players? Or is your performance team well-coordinated?

Can you explain how you plan the training content for national team camps and tournaments? How do you structure the workload—volume, frequency of sessions, balance between handball and physical training, etc.?

According to your philosophy, players are expected to take responsibility for their own development, which is a significant demand. Do many players struggle to navigate that landscape effectively?

Let’s now shift the focus more specifically to X. What made you select her for the national team? Had you followed her development closely in the years leading up to her selection?

X began playing in the top division at age 16 and was already a key player in her club that season. How rare is it for a 16-year-old to make such an impact at senior level?

Can you describe the range of development pathways on the national team? X made an early breakthrough and was dominating in the top league already at 16–17. She also played on nearly all the youth national teams. Other players on your team, however, were late bloomers and eventually became just as good. What are your thoughts on these different trajectories, and how do you approach them?

It took X a few seasons to earn a regular place on the national team. Did you have a long-term plan for her development? In her first championship, you sent her home after the preliminary rounds and brought in another player. How did she handle those challenges? Did you ever doubt that she would become a regular on the national team?

You have often selected young, talented players and shown great patience with them. Is this a conscious part of your coaching philosophy?

What specific areas has X worked on over the years to become a better player on the national team—whether technical, tactical, physical, mental, interpersonal, or social?

You eventually chose X the captain of the national team. Why? Coach 2 told me that he didn’t see that coming, as he had previously experienced her as somewhat conflict averse. What were your considerations in making that decision?

When I spoke with X, she mentioned that her husband feels the Norwegian national team holds a uniquely high status. In other countries, clubs are the players’ primary employers, and careers revolve mostly around club commitments. But for Norwegian female players, as he sees it, their careers are centered around the national team, with most decisions made in light of it. Do you have any thoughts on why and how the national team has earned such status?

Can you describe how you’ve collaborated with X’s club coaches over the years? Has the cooperation generally been positive, or have there been challenges?

If you were to summarize X’s career, what would you say are the key reasons why she developed into one of the world’s best players?

Throughout her career, X has been remarkably free of injuries, despite her fearless playing style. Do you have any thoughts on why she has managed to stay relatively injury-free?

That’s all the questions I have. Is there anything you would like to add—something I haven’t asked about, but you feel is important to mention?
